# Diabetes- versus smoking-related thrombo-inflammation in peripheral artery disease

**DOI:** 10.1186/s12933-023-01990-6

**Published:** 2023-09-21

**Authors:** T. Alnima, R. I. Meijer, H. M.H. Spronk, M. Warlé, H. ten Cate

**Affiliations:** 1 Department of Internal Medicine, Section of Vascular Medicine, Radboud University Medical Center, Nijmegen, The Netherlands; 2 Department of Internal Medicine, Section of Diabetology, Radboud University Medical Center, Nijmegen, The Netherlands; 3 Thrombosis Expertise Center, Heart and Vascular Center, Maastricht University Medical Center+, Maastricht, The Netherlands; 4 Department of Vascular Surgery, Radboud University Medical Center, Nijmegen, The Netherlands

**Keywords:** Peripheral artery disease, Diabetes mellitus, Smoking, Thrombosis, Inflammation

## Abstract

Peripheral artery disease (PAD) is a major health problem with increased cardiovascular mortality, morbidity and disabling critical limb threatening ischemia (CLTI) and amputation. Diabetes mellitus (DM) and cigarette smoke are the main risk factors for the development of PAD. Although diabetes related PAD shows an accelerated course with worse outcome regarding complications, mortality and amputations compared with non-diabetic patients, current medical treatment does not make this distinction and includes standard antiplatelet and lipid lowering drugs for all patients with PAD. In this review we discuss the pathophysiologic mechanisms of PAD, with focus on differences in thrombo-inflammatory processes between diabetes-related and smoking-related PAD, and hypothesize on possible mechanisms for the progressive course of PAD in DM. Furthermore, we comment on current medical treatment and speculate on alternative medical drug options for patients with PAD and DM.

## Introduction

Peripheral artery disease (PAD) is a serious public health problem associated with high risk of cardiovascular complications and mortality [1]. The overall prevalence of PAD in people aged 25 years and older is 5.56% with the prevalence increasing consistently with age [2]. PAD is the important risk factor for lower-extremity amputation, especially in diabetic patients with chronic foot ulcers [2, 3]. It is widely accepted that the cardiovascular event rates in patients with PAD and diabetes mellitus (DM) are higher than in those without DM, suggesting a more progressive course of the disease [4]. In a systematic review examining the interrelationship between DM and PAD, the prevalence of PAD is higher in diabetic versus non-diabetic populations, exceeding 50% in patients with DM and foot ulceration. Additionally, patients with DM had worse outcome regarding perioperative complications, amputations, and mortality compared with non-diabetic patients [5].

The pathophysiology of PAD in DM is believed to be similar to that in non-diabetic patients [6]. Nevertheless, the affected vascular beds differ between diabetic and non-diabetic patients. The distribution of the affected peripheral arteries in diabetic patients with PAD is often more distal, with common involvement of the tibial and peroneal arteries, whereas PAD in smokers mainly affects the proximal arteries [7]^,^ [8]. Diabetic patients with PAD commonly show symmetrical and multi-segmental stenoses, which are even present in the collateral vessels [3]. Apart from worse lower extremity function, diabetic patients with PAD are at increased risk for sudden arterial thrombosis and ischemic ulceration of the lower limbs [4].

The progressive natural history of PAD in DM may suggest other pathophysiological processes next to atherosclerosis. Inflammation, hypercoagulability and blood viscosity are known to contribute to the initiation and propagation of atherosclerosis and may aggravate the progress of PAD [9–11]. However, little is known about the magnitude and mechanisms of the accelerated thrombo-inflammatory process in patients with PAD and DM versus those without DM.

In this narrative review, we present current knowledge on the pathophysiological differences and similarities in PAD between patients with and without DM. Our aim is to find out whether the accelerated process of PAD in people with DM is associated with higher thrombo-inflammatory responses compared with PAD in people without DM, mainly focusing on patients with type 2 DM. Because the majority of diabetic patients have type 2 DM, this review will mainly focus on this specific subtype of DM.

### Pathogenesis of diabetes- and smoking-related pad

In order to put the contribution of DM to atherosclerosis, inflammation and thrombosis in PAD into perspective, we were interested in the pathology of PAD associated with smoking, which is the most common risk factor for PAD [12]. To the best of our knowledge, there are no clear data directly comparing diabetic patients with PAD versus smokers with PAD. Such distinctive data are also hard to obtain due to the frequent co-existence of combined risk factors. The co-existence of PAD in smoking diabetic patients varies widely depending on socio-economic status and geographic region and has been demonstrated to be roughly between 30 and 80% in several endovascular studies [3, 13–15].

**Fig. 1:**
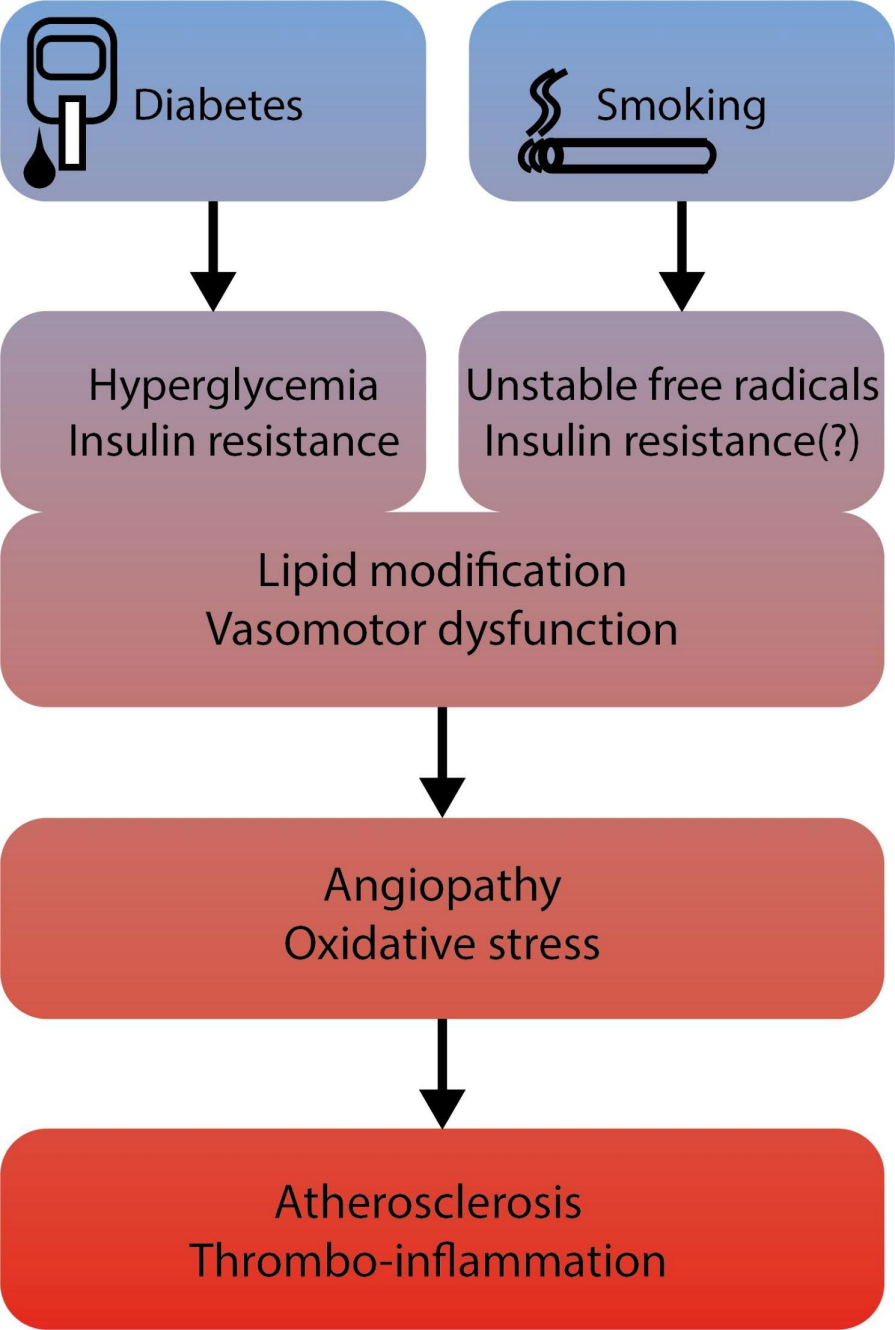
Pathological pathways leading to angiopathy and peripheral artery disease in diabetes mellitus and cigarette smoking. DM: diabetes mellitus

Cigarette smoke induces vasomotor dysfunction, inflammation and modification of lipids. The mechanisms by which smoking accelerates vascular dysfunction are manifold and challenging because smoke contains over 4000 different chemicals [16]. In contrast, PAD in DM is the result of angiopathy due to the abnormal metabolic state characterized by chronic hyperglycemia, insulin resistance and release of excess free fatty acids. In both cases, oxidative stress constitutes an important trigger for the emergence of microvascular and macrovascular complications (Fig. 1). In DM, overproduction of mitochondrial ROS (reactive oxygen species) leads to the activation of pathways involved in the pathogenesis of vascular complications [17]. Furthermore, ROS inactivate critical defensive enzymes, like eNOS and prostacyclin synthase. Consequently, increased intracellular ROS cause defective angiogenesis, activation of proinflammatory pathways and chronic epigenetic changes which persistently stimulate a pro-inflammatory state even after achieving normoglycemia (‘hyperglycemic memory’) [18]. Cigarette smoke contains a number of highly unstable free radicals, which enhance ROS production, resulting in an imbalance between production and detoxification of these species, and dampening the antioxidant status [19].

### Plaque characteristics in diabetes- and smoking-related pad

PAD is mainly caused by atherosclerosis and associated thrombotic occlusion of one or more peripheral arteries [20]. Plaque composition is heterogeneous in different vascular beds, which may be related to various hemodynamic forces, elastic and muscular components of arterial wall and the triggers for initiation and progression of atherosclerosis [21]. Acute limb ischemia, characterized by sudden drop in limb perfusion, is often caused by arterial emboli or in situ thrombi due to plaque progression and complication [22]. Soor et al. examined 1305 arterial segments from patients who underwent lower limb amputation, mainly due to chronic limb ischemia [23]. The severity of medial calcification was higher in diabetic versus non-diabetic patients, with a greater degree of atherosclerotic narrowing. In another study, 239 arteries from patients with mainly chronic limb ischemia, and the majority with DM, were examined and the pathological characteristics were recorded for femoral, popliteal, and infrapopliteal arteries [24]. Atherosclerotic plaque composition was more frequently observed in femoro-popliteal arteries compared with infrapopliteal arteries. Chronic luminal thrombi were more frequently present in arteries with insignificant atherosclerosis, especially in infrapopliteal arteries. The majority of atherosclerotic plaques showed ≥ 70% stenosis, due to intimal thickening, fibroatheroma, fibrocalcific lesions or restenosis, or due to luminal thrombi. In 73% of the arteries, was the presence of in situ thrombi contributing to luminal stenosis. Finally, van Haelst et al. analyzed plaque characteristics in patients with and without DM who underwent endarterectomy of the femoral or iliac artery [13]. Remarkably, there were significantly more smokers in the non-diabetic group compared with the group with DM, 46% versus 34% respectively. Pathologic sections from patients with DM showed more calcified plaques compared with those without DM. Reports on the relationship between cigarette smoking and plaque characteristics are very limited. Kumagai et al. showed that smoking is associated with lipid-rich plaques in patients undergoing percutaneous coronary intervention, which was explained by smoking induced insulin resistance or hyperinsulinemia [25].

In summary, not only plaque localization, but also plaque composition seems different between diabetic and non-diabetic patients (e.g. smokers) with PAD. Generally, diabetic patients show more distal or infrapopliteal pathology, have a greater degree of atherosclerotic narrowing, medial calcification, and in situ thrombi.

### Thrombo-inflammation in diabetes- and smoking-related pad

To the best of our knowledge, there are no studies on the rate of thrombo-inflammation in PAD comparing diabetic- and non-diabetic patients or smokers. Below we summarize current knowledge on the thrombo-inflammatory processes in diabetes-related and smoking-related PAD.

### Endothelial dysfunction

Loss of antithrombotic and anti-inflammatory functions of endothelial cells leads to dysregulation of complement, coagulation, platelet activation, and leukocyte recruitment. Impairment of endothelial vasodilation is an early manifestation of atherosclerosis. Cigarette smoke impairs endothelium-dependent vasodilation in macrovascular beds in humans by reducing nitric oxide (NO) availability [26, 27]. Apart from causing vasodilation, NO plays a role in regulation of inflammation, leukocyte adhesion, platelet activation and thrombosis. Smoking also modifies lipid profiles in a proatherogenic manner, with free radicals and oxidants present in cigarette smoke causing pro-oxidative lipid modification and atherogenesis [28]. The exact components of cigarette smoke and the mechanisms responsible for endothelial dysfunction have not been clearly elucidated yet.

Hyperglycemia triggers vascular damage by inducing a disbalance between NO bioavailability and oxidative stress by accumulation of ROS resulting in endothelial dysfunction. Additionally, several other cellular mechanisms are induced by hyperglycemia resulting in vascular damage; enhanced production of advanced glycation end products (AGEs), augmented polyol and hexosamine flux and activation of protein kinase C, resulting in increased oxidative stress from ROS, expression of procoagulant activity, inflammatory cytokines and growth factors with activation of nuclear factor ĶB (a transcription factor for activation of variety of pro-inflammatory genes) [17]. Consequently, the production of the potent vasodilator and anti-inflammatory agent NO is decreased.

### Platelet hyperreactivity

Platelet hyperreactivity is involved in a wide variety of clinical settings including PAD and DM, even during antiplatelet therapy [29–33]. One of the prothrombotic effects of smoking is alteration in platelet function. Exposure to smokers’ serum causes hyperaggregation in isolated platelets from non-smokers [34]. Interestingly, antithrombotic therapy seems to be more effective in smokers than in non-smokers. A recent meta-analysis on the impact of smoking on platelet ADP-P2Y_12_ receptor inhibitors shows a stronger platelet inhibition in the smoking group, even in smokers with DM versus those without DM [35, 36]. The lower residual platelet reactivity observed in smokers may explain variations in clinical outcomes in PAD subgroups, especially in people with DM.

In damaged endothelium, the production of antiaggregatory molecules (e.g. NO) is impaired, but platelets from patients with DM have a reduced sensitivity to anti-aggregants (e.g. prostacyclin) [37, 38]. Additionally, platelets of patients with type 2 DM are characterized by increased expression of activation markers (CD31, CD49b, CD62P and CD63) [39]. Hyperglycemia is associated with increased platelet reactivity by several mechanisms. First, hyperglycemia results in glycation of platelet surface proteins, which impairs fluidity of the membrane with increased platelet adhesion. Second, hyperglycemia induces protein kinase C (PKC) activity triggering platelet activation [40]. Furthermore, increased protein levels of von Willebrand factor (vWF), reflecting endothelial cell damage, have been described in DM, thereby promoting platelet adhesion [41]. Lastly, during combined hyperglycemia and hyperinsulinemia platelets increase expression of CD40L, which interacts with monocytes and endothelial cells inducing a cascade of inflammatory responses including tissue factor (TF) expression [42].

The effect of hyperinsulinemia on platelets is complex and disparate between healthy and insulin resistant individuals. Platelets retain an insulin receptor, which upon activation results in reduced platelet responses to several agonists like ADP, collagen, thrombin and arachidonate [43]. Other features of DM, like obesity, may induce insulin resistance contributing to platelet hyperreactivity [44]. Finally, oxidative stress increases intraplatelet calcium release upon activation, thereby amplifying platelet aggregation [45].

### Hyperinflammation

Increased concentrations of C-reactive protein (CRP), interleukin-6 (IL-6) and tumor necrosis factor alpha (TNF-α) have been found in smokers [46]. Cigarettes also induce the production of several chemokines and proinflammatory cytokines enhancing leukocyte recruitment. Indeed, levels of soluble VCAM-1, ICAM-1, E-selectin were higher in smokers [47]. These factors not only promote the adhesion of monocytes to endothelial cells, but also drive monocytes to pass the endothelium, enter the tissues, and alter their phenotype. In addition, some components of cigarette smoke activate PKC, resulting in increased expression of monocyte adhesion ligand CD11b, further enhancing monocyte adhesion to endothelium [48]. Recent data support the participation of neutrophils in the proinflammatory and prothrombotic state in atherosclerosis. Neutrophil activation enables the release of neutrophil extracellular traps (NETs), a form of programmed cell death, involving DNA, histones and granular enzymes, which are not only capable of ensnaring and killing pathogens, but also promote a proinflammatory and prothrombotic state [49]. NETosis, the process of NET formation, has been detected in patients with smoking-related chronic pulmonary inflammation [50]. However, little is known about the extent of NETosis in smoking-related PAD. Nevertheless, limited data demonstrate the presence of citrullinated histone-3, a marker of NETosis, in thrombi from patients with PAD, including smokers [51–53].

Metabolic abnormalities found in DM are thought to either induce an inflammatory response, or to be exacerbated by or associated with inflammation [54]. Excessive levels of glucose and free fatty acids induce stress in insulin-sensitive tissues resulting in the release of several cytokines and chemokines including CRP, fibrinogen, plasminogen activator inhibitor (PAI), IL-1β, TNF-α, CC-chemokine ligand 2 (CCL2), CCL3, CXC-chemokine ligand 8 [55, 56]. Immune cells are then further recruited to contribute to tissue inflammation. NETs are released from activated neutrophils in response to interleukins and ROS, which further promote platelet adhesion and aggregation, bind fibrinogen, and thrombin generation [57]. Growing evidence supports an association of increased circulating markers of NETosis in type 2 DM and hyperglycemia, with impaired wound healing [58–60]. Importantly, macrophages in adipose tissue produce a significant portion of the inflammatory factors that are upregulated by obesity, a common factor in type 2 DM [61]. The degree of inflammation may vary within individuals and between tissues and does not necessarily reflect the degree of systemic inflammation. Potential mechanisms involved in this inflammatory response are hypoxia and cell death in the expanding adipose tissue, activation of the inflammation- and stress-induced kinases IκB kinase-β (IKKβ) and JUN N-terminal kinase (JNK), which further stimulate inflammation and insulin resistance [62].

The role of the complement system in initiation and propagation of atherothrombosis in diabetes-related and smoking-related PAD is not well studied. Nevertheless, complement activation in the pathogenesis of cardiovascular disease is well described, from the earliest signs of endothelial dysfunction, followed by progression to the formation of atherosclerotic plaques and vascular thrombus, with accompanied proinflammatory and procoagulant changes [63, 64].

### Hypercoagulation

Cigarette smoke induces a prothrombotic and antifibrinolytic state. Exposure of human monocytes/macrophages to cigarette smoke induces cell surface TF display and generation of procoagulant microvesicles [65]. Moreover, circulating vWF and TF are increased in smokers [66]. In addition, endothelial cell derived tissue factor pathway inhibitor (TFPI), a potent regulator of TF-factor VIIa-factor Xa-dependent activation pathway, is decreased in smokers. This prothrombotic alteration in TF/TFPI could be mediated by decreased NO bioavailability [67]. Also, plasma fibrinogen, responsible for augmenting platelet aggregation by linking glycoprotein IIb/IIIa receptors between platelets and providing thrombus support by a matrix of cross-linked fibrin, is higher among smokers [68]. Finally, factor XIII (FXIII), which is responsible for clot stabilization, is elevated in smokers [69].

Parameters of increased coagulability have also been found in DM. Hyperglycemia, particularly in combination with hyperinsulinemia, leads to a procoagulant state by increased levels of TF, decreased factor VII/VIIa and increased factor VIII and prothrombin fragment F1.2 [70]. This is of particular importance in patients with poor glycemic control. Platelet dependent thrombin generation is higher in patients with poor glycemic control than in healthy subjects and patients with well-controlled DM [71]. Several mechanisms are involved in this procoagulant state. Hyperinsulinemia and hyperglycemia directly stimulate TF transcription in monocytes [72]. Generation of AGEs and other glycated proteins during hyperglycemia, as well as ROS upregulate TF production through activation of the NF-kB inflammatory pathway [73, 74]. In addition, increases in factors VIII, IX and XI are observed in increased fasting plasma glucose [75]. Finally, hyperfibrinogenemia is a common finding in diabetes, and may be explained by enhanced fibrinogen production in states of hyperglycemia and insulin resistance [76]. Enhanced oxidative stress and glycation of fibrinogen also alter the structure of fibrinogen and fibrin, resulting in fibrinolysis-resistant clots [77].

A straightforward result of elevated prothrombotic activity is increased thrombin generation. However, the relation between plasma thrombin generation potential and atherothrombotic disease manifestation is less obvious and appears inconclusive from earlier reports [78, 79]. Furthermore, very little is known about thrombin generation in PAD in smokers and diabetic patients [80, 81].

### Hypofibrinolysis

Timely dissolution of a clot is of great importance to prevent pathological propagation of a thrombus. The endothelial cell lining forms a major source of both fibrinolytic (tissue plasminogen activator, tPA) and antifibrinolytic (PAI-1) factors. Fibrinolysis is mediated by plasmin, which is activated from plasminogen by tPA [66]. On the other hand, tPA is inhibited by PAI-1. Clot lysis is reduced in venous clots from smokers after addition of tPA [82]. Chronic smoking is associated with fibrinolytic alterations mainly by elevated plasma PAI-1 [83]. Additionally, infusion of substance P in smokers and non-smokers caused inhibition of tPA release in smokers, resulting in reduced fibrinolytic capacity in chronic smoking [84].

A marked fibrinolytic impairment has been found in type 2 DM due to high concentrations of PAI-1 [85]. In addition, lower clot permeability, longer clot lysis time, and higher maximum D-dimer levels were observed in patients with diabetes for longer than 5 years and those with HbA1c > 6.5% [86]. On a cellular level, hyperglycemia and hyperinsulinemia increase the expression of PAI-1 by vascular smooth muscle cells in vitro, thereby reducing the activity of t-PA and fibrinolytic potential [87]. In addition, posttranslational modifications of both fibrinogen and plasminogen, induced by metabolic abnormalities in DM, are implicated in hypofibrinolysis. Glycation of α2-antiplasmin is one of the possible links to compromised fibrinolysis [68].

**Fig. 2:**
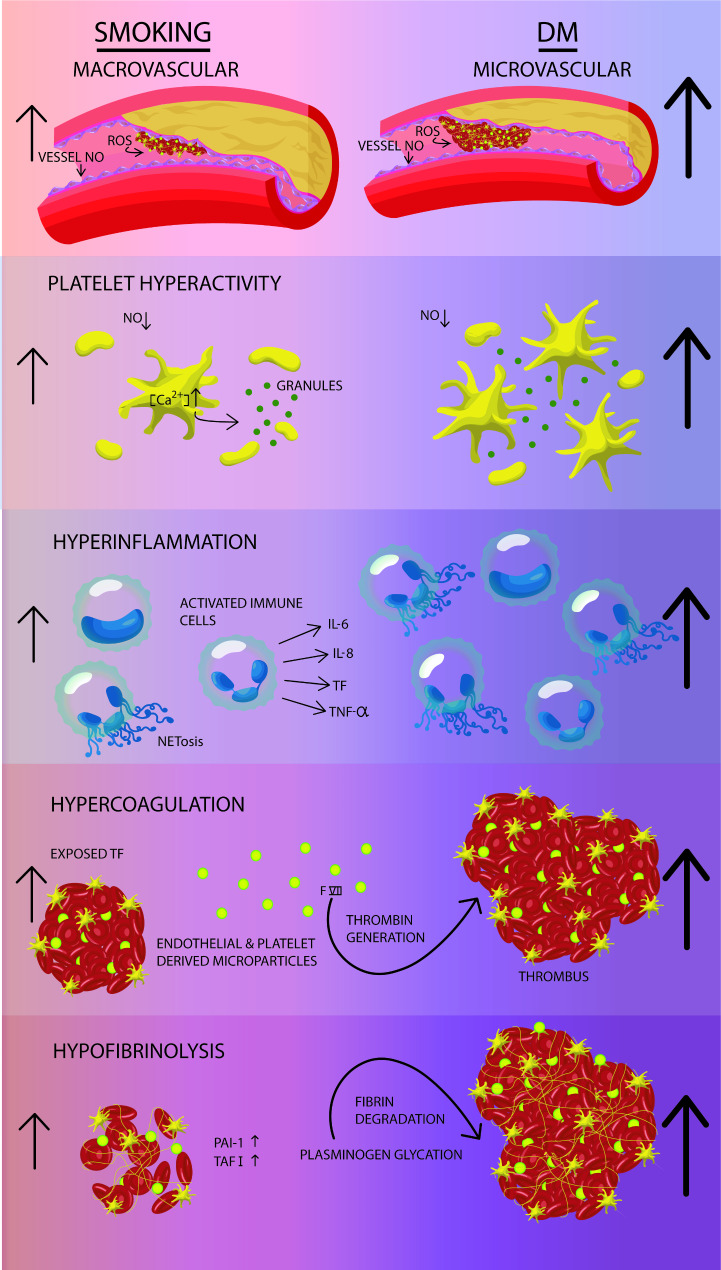
Hypothetical image of the increasingly thrombo-inflammatory processes behind the course of peripheral artery disease in diabetes mellitus compared with cigarette smoking. DM: diabetes mellitus. NO: nitric oxide. ROS: reactive oxygen species. TF: tissue factor. TNF-α: tumor necrosis factor-α. PAI-1: plasminogen activator inhibitor-1. TAFI: thrombin activatable fibrinolysis inhibitor

In summary, we reviewed the pathophysiology of PAD in DM and put that next to PAD in smokers. It is striking that current insights on the pathophysiology of PAD show little difference between DM-related and smoking-related PAD. Patients with DM as well as smokers with PAD have signs of endothelial dysfunction with increased inflammatory and prothrombotic activity. Nevertheless, it is generally accepted that PAD in DM is associated with worse prognosis than PAD without DM. Noticeably, smokers seem to be better responders to antithrombotic therapy than non-smokers. Yet, this is insufficient to explain the more aggressive course of PAD in patients with DM. Interestingly, evidence on differences in magnitude of the thrombo-inflammatory responses between diabetic and non-diabetic patients with PAD is still lacking. In our opinion, it is likely that patients with PAD and diabetes express a greater inflammatory reaction with higher prothrombotic tendency when compared with smoking-related PAD due to several serious metabolic disturbances in DM (Fig. 2). If this is true, it means that current medical treatment for diabetic patients with PAD is suboptimal and needs to be re-evaluated. Indeed, many previous studies have demonstrated worse outcome for PAD in diabetic patients despite standard of care treatment. Obtaining new insights into the magnitude of the inflammatory and prothrombotic status between diabetic and non-diabetic patients with PAD may offer more accurate and other therapeutic strategies for this very high risk cardiovascular group.

### Therapeutic options in pad and diabetes mellitus

Current medical treatment used in PAD is selected to target atherothrombotic pathways and supporting data stem mainly from studies in patients with coronary or cerebrovascular disease. Despite optimal medical treatment, a significant proportion of patients, especially those with diabetes, will continue to suffer from cardiovascular events, including major amputations [88]. Therefore, further optimalization of medical treatment in PAD and DM is still required.

### Antiplatelet drugs

Thus far, no clear evidence supports any form of antithrombotic therapy in patients with asymptomatic PAD and DM [89, 90]. In symptomatic PAD, antithrombotic therapy provided a proportional reduction of 23% in serious adverse events [91]. Strikingly, among 4961 patients with DM analyzed in this meta-analysis, antiplatelet therapy was associated with only a 7% proportional reduction in serious vascular events with a higher overall incidence of vascular events in patients with DM. However, the majority of diabetic patients had generally no history of myocardial infarction or stroke and the benefit was considered consistent (although 22% reduction observed overall) [91, 92]. It is still of interest to consider whether there is good evidence that proportional risk reduction is equal in different patient categories and to assume that patients with DM have less net benefit of antithrombotic therapy due to their extensive metabolic disturbances and greater residual risk.

Aspirin is still the most often used antiplatelet agent in PAD. Although the CAPRIE trial demonstrated that clopidogrel versus aspirin reduced the relative risk of major adverse cardiovascular events by 8.7% in favor of clopidogrel, yet the absolute risk reduction was small with a number needed to treat (NNT) of 200, without difference in amputation rate between treatment groups [93]. Subgroup analysis showed even greater risk reduction in patients with PAD, but the study was not powered to provide a definitive answer for this subgroup. Bhatt et al. performed additional subanalyses in the CAPRIE group with DM and observed a greater benefit of clopidogrel versus aspirin in reducing recurrent ischemic events [94]. The annual event rate was 15.6% in the clopidogrel group and 17.7% in the aspirin group with an absolute risk reduction of 2.1% and a number needed to treat of 48. There was no additional subdivision in the group of diabetic patients with PAD in that study, and no data about the severity and duration of diabetes. A systematic review reported that aspirin, ticlopidine, and ticagrelor or clopidogrel use as monotherapy (or in combination with aspirin) were effective in reducing major cardiovascular events in patients with PAD, with clopidogrel showing the best safety profile [95]. On the other hand, ticlopidine, the PAR-1 antagonist vorapaxar and DAPT (dual antiplatelet therapy) increased bleeding risk despite their beneficial effect on reducing major cardiovascular events.

Another important aspect of antiplatelet use in PAD, is the emerging data on suboptimal responsiveness of patients with DM to most prescribed antiplatelet agents [96]. This reduced platelet inhibition appeared to be related to obesity and higher platelet turnover [97]. Therefore, a twice-daily aspirin regime has been suggested and reported to prolong the platelet-derived thromboxane A2 inhibition [97, 98]. However, these findings need to be further established in randomized trials, specifically in patients with PAD and DM. Reduced platelet responsiveness to other antiplatelets have also been reported in patients with DM. The fraction of poor responders appeared higher in diabetic patients treated with clopidogrel and prasugrel, due to reduced generation of their active metabolites [99].

### Anti-inflammatory drugs

Existing cardiovascular drugs, e.g. statins, yield secondary benefits by reducing inflammation. Nevertheless, the need for other therapeutic options to reduce the residual cardiovascular risk is still necessary. Targeted anti-inflammatory treatment is a promising strategy in the treatment of PAD.

Methotrexate and colchicine are anti-inflammatory agents with mechanisms of action that are not fully understood, but may be of value in the treatment of cardiovascular disorders. Especially colchicine seems an attractive agent, considering its ability to interfere with cytoskeleton of cells and altering expression of proteins expressed by leukocytes, platelets and endothelial cells during inflammatory processes [100]. However, low-dose methotrexate did not reduce levels of inflammatory markers and did not result in fewer cardiovascular events compared with placebo in patients with stable atherosclerotic disease and DM or metabolic syndrome [101]. On the other hand, the risk of cardiovascular events was significantly lower among patients with stable coronary disease who received colchicine compared with placebo, regardless of DM status, and resulted in 31% of risk reduction and a NNT of 35 [102]. Data from randomized trials of the efficacy of colchicine in PAD and DM are awaited.

TNF-α is a critical regulator of vascular inflammation. However, no data from randomized trials are available on the use of anti-TNF-α in patients with cardiovascular disease, or PAD and DM in particular. Both IL-1α and IL-1β are highly expressed in atherosclerotic lesions promoting recruitment of leukocytes, oxidative stress and pro-coagulant activity. Limited data support the use of anakinra, an IL-1 receptor antagonist, in patients with acute coronary syndrome, with no randomized trials in PAD and DM [103]. Inhibition of IL-1β by canakinumab, reduced the rates of nonfatal myocardial infarction, nonfatal stroke, or cardiovascular death compared with placebo in patients with prior myocardial infarction and high sensitivity CRP ≥ 2 mg/L, with consistent effects regardless of DM [104, 105]. Nevertheless, there is no data on the use of canakinumab in PAD and DM. Tocilizumab, an IL-6 targeting antibody, used in rheumatologic disorders, improves endothelial function and reduces aortic stiffness in patients with rheumatoid arthritis [106]. Thus far, no studies were specifically directed to PAD and DM regarding the use of tocilizumab. Furthermore, IL-6 and other cytokines are able to activate the JAK2/JAK2-STAT3/STAT3 pathway in fibroblasts and endothelial cells, which facilitates the process of endothelial damage, atheroma formation and atherosclerosis. Therefore, it is hypothesized that JAK2 inhibition may have potential benefit in preventing adverse cardiovascular events [107].

Interest in therapeutic compounds that specifically block NET formation and inhibit their detrimental effects in atherothrombotic disease is growing. Injection of deoxyribonuclease (DNase), which degrades NETs by hydrolysis of the DNA backbone, reduced lesion size and pro-inflammatory cytokines in atherosclerotic mice [108]. Inhibition of peptidylarginine deiminase, a critical enzyme for efficient uncoiling of chromatin in NETosis, reduced atherosclerotic lesion and delayed time to carotid artery thrombosis in atherosclerotic mice treated with CI-amidine [109]. None of these compounds have been tested in PAD or DM yet. Interestingly, colchicine and certain antibiotics like azithromycin are also able to inhibit NETosis but have not been tested in this specific purpose and setting before [100, 110].

### Anticoagulation

The emergence of direct-acting oral anticoagulants (DOACs), specifically rivaroxaban, has increased treatment options for patients with arterial vascular disease, including PAD and DM. The relative high percentage of intraluminal thrombi in patients with PAD and DM, and overall elevated D-dimer levels in PAD provide mechanistic support to the observed benefit of the COMPASS trial regime, illustrating the importance of hypercoagulability as a contributing factor in disease progression. The COMPASS trial tested rivaroxaban 2.5 mg twice daily in combination with aspirin, rivaroxaban 5 mg twice daily, or aspirin once daily, in patients with chronic coronary artery disease or PAD. After a mean follow up of 23 months, the primary composite outcome of cardiovascular death, stroke or myocardial infarction, occurred in fewer patients in the rivaroxaban plus aspirin group than in the aspirin group alone [111]. However, major bleeding events occurred more frequently in the rivaroxaban plus aspirin group. Subgroup analysis showed no difference in outcome between patients with or without DM. The VOYAGER PAD trial evaluated the efficacy of rivaroxaban plus aspirin in the reduction of major cardiovascular and limb ischemic vascular outcomes in patients with PAD undergoing lower-extremity revascularization. After 3 years of follow up, a significant reduction of acute limb ischemia, major amputation, myocardial infarction, ischemic stroke or cardiovascular death was observed in the rivaroxaban plus aspirin group compared with aspirin alone [112]. Major bleeding was significantly higher in the rivaroxaban plus aspirin group. A subgroup analysis of VOYAGER showed that efficacy of rivaroxaban was consistent with the overall group regardless of the existence of DM (ESC 2021, VOYAGER PAD Rivaroxaban in symptomatic PAD with and without comorbid diabetes).

Other antithrombotic alternatives containing anticoagulants were tested before. In the WAVE trial, patients with PAD (27% DM) were assigned to a combination of vitamin K antagonist with the antiplatelet agents aspirin, ticlopidine or clopidogrel. The combination therapy was not more effective than antiplatelet therapy alone in preventing major cardiovascular events, but was associated with more life-threatening bleeding events [113]. Among DOACs, rivaroxaban attracted most of the interest in PAD due to its postulated pleiotropic effects in vascular protection [114]. Other DOACs have been studied less intensive in atherosclerotic disease. Edoxaban plus aspirin demonstrated a similar risk for major and life-threatening bleedings events compared with aspirin plus clopidogrel in patients with PAD following endovascular treatment [115]. Incidence of restenosis/re-occlusion was not statistically different between study groups. The results of the AGRIPPA study, a trial exploring the efficacy and safety of low dose apixaban plus aspirin compared with clopidogrel plus aspirin, are still awaited [116]. Dabigatran binds to the active site of thrombin, thereby preventing thrombin-induced activation of factors V, VIII, XI, fibrin formation, and thrombin-mediated platelet activation and aggregation [117]. From a physiological perspective, it seems interesting to investigate whether dabigatran would be of greater benefit when combined with aspirin, compared with rivaroxaban plus aspirin in reducing cardiovascular events in patients with PAD and DM, considering the additional potential effect of dabigatran on platelet function and fibrinolysis.

### Fibrinolytic drug treatment

Current fibrinolytic therapy consists of thrombolysis with recombinant tPA or streptokinase and is mainly used in cases with acute limb ischemia. Thus far, no evidence is in favor of either initial thrombolysis or surgery in terms of limb salvage, amputation or death [118].

Less is known about the use of other components of the fibrinolytic pathway as a target for therapy in cardiovascular disease. PAI-1, originally recognized for its role in fibrinolysis, is nowadays more often linked to other complex pathophysiological processes including atherosclerosis and metabolic disorders [119]. The crucial role of PAI-1 in the fibrinolytic system is to inhibit tPA and urokinase-type plasminogen activator (uPA), resulting in attenuation of plasminogen activation and fibrin degradation. The PAI-1 plasma concentration is low, but the major source is located within platelet α-granules [120]. PAI-1 is also expressed by other cell types including megakaryocytes, adipocytes and endothelial cells. Increased levels of PAI-1 are associated with thrombotic events [119]. Plasma PAI-1 is also associated with onset of type 2 DM and could be an important clinical marker for development of future cardiovascular disease [121, 122]. Even though several PAI-1 inhibitors have been developed, none of them have been approved to date to use in humans. This is surprising, as it physiologically seems an attractive target with a safe profile considering the mild-to moderate bleeding tendency in homozygous PAI-1 deficient individuals [123]. Interestingly, a few PAI-1 inhibitors are currently under investigation, emphasizing the important role of PAI-1 in various pathophysiological processes including cardiovascular disease [124].

Another essential player in fibrinolysis is the rapid-acting plasmin inhibitor α2-antiplasmin (α2AP). It is primarily synthesized in the liver and upon its release in the circulation, it becomes enzymatically modified affecting its fibrin-crosslinking and plasmin binding abilities [125]. Experimental studies suggest that α2AP inhibition may offer a novel strategy for preventing thrombosis and dissolution of thrombi [126]. Novel α2AP inactivation compound is currently tested in a phase 2 trial in subjects with intermediate-risk pulmonary embolism (ClinicalTrials.gov, NAIL-IT Trial). Thus far, we found no planned studies with α2AP inhibition in PAD.

Finally, inhibition of FXIII may offer a novel treatment target in cardiovascular disease. FXIII is present in monocytes and macrophages, which play a major role in the process of atherogenesis [127]. Elevated levels of FXIII have been shown to confer an increased risk of PAD, mainly in women [128]. Therefore, FXIII inhibition may be an interesting treatment target, although bleeding side effects may be foreseen due to its impact on fibrin cross linking. Indeed, congenital FXIII deficiency is associated with a variable degree of bleeding ranging from mild to life threatening bleeding events [129]. Despite the existence of different FXIII inhibitors, these are currently not available for medical application [130].

### Statins

Although statins are mainly prescribed for their lipid lowering effect in atherosclerotic disease, they are currently also recognized as anti-inflammatory agents with emerging evidence that their benefit may also involve antithrombotic effects. One of the primary mechanisms responsible for the anti-inflammatory properties, is the upregulation of endothelial vascular protective functions, mediated by endothelial cell nitric oxide synthase and a subsequent rise in NO bioavailability [131]. The antithrombotic effects may involve inhibition of platelet thromboxane A2, platelet isoprostane formation, and inhibition of fibrinogen receptor gpIIIa on platelet-derived microparticles, as well as impaired thrombin generation by downregulation of TF or upregulation of thrombomodulin [132]. Statin therapy was associated with significant reduction in cardiovascular mortality, risk for amputation, or loss of patency after endovascular treatment, with improved outcomes at higher dose [132]. However, very little is known about anti-inflammatory and anti-thrombotic effects of statins in PAD with and without DM or diabetic patients versus smokers with PAD.

To sum up the findings on medical therapy in PAD and DM, it basically consists of statins and antiplatelet therapy, preferably clopidogrel, irrespective of the main driver for the initiation and propagation of PAD. Patients with DM seem to respond less well to antiplatelet therapy, while smokers generally respond adequately to antiplatelet drugs (smoker’s paradox). The question is are those ‘responders’ mainly the smokers without diabetes and the ‘non-responders’ the ones with DM. Reviewing the data comparing smokers versus non-smokers and antiplatelet responsiveness, provide often no information or correction for the concomitant existence of DM and cigarette smoke, which makes is hard to interpret these data. In our opinion and after analyzing several studies, non-smokers with atherosclerotic disease (e.g. PAD) tend to have more often (pre-)DM [35, 133]. Despite the lack of direct head-to-head comparison of responsiveness of diabetic patients versus smokers to antiplatelet therapy, increasing the dose of antiplatelet therapy in patients with DM may be an option to improve platelet inhibition and may be an interesting avenue for future studies. Dual pathway inhibition by aspirin plus low dose rivaroxaban provided a similar degree of benefit on cardiovascular end points in patients with and without DM. However, given the higher baseline risk and residual risk in diabetic patients, there is need for more effective approach in this population. Thus far, there is no standard role for anti-inflammatory therapy in the treatment of PAD in DM, despite the good results obtained with colchicine in coronary artery disease. The results of the effect of colchicine in PAD are still awaited. However, from a pathophysiological point of view and regarding the hyperinflammatory state in DM and PAD, it is reasonable to target the inflammatory system as a therapeutic strategy. Colchicine seems an attractive option for this purpose, but future work is needed to evaluate the most suitable anti-inflammatory drug treatment in this specific setting. Considering the use of fibrinolytic drugs in PAD and DM may offer new treatment options. There is little movement going on regarding the use of other fibrinolytic drugs than alteplase or urokinase. A novel α2AP inhibitory compound is currently tested for the use in venous thrombo-embolism. Other components of the fibrinolytic system are also worth investigating as a target for treatment in PAD, like FVIII or PAI-1 inhibition. A final important remark, is the challenge to select the right patient for the most optimal treatment combination, which is in our opinion a dynamic process requiring adjustment to the clinical presentation and progression of the disease during follow up.

## Conclusion

PAD shows greater progression in patients with DM resulting in more major limb complications and other cardiovascular events, as compared to non-diabetic PAD. The cause of the aggravated course of PAD in DM remains unexplained. Based on current literature review, we conclude that both smoking-related and DM-related PAD have an increased thrombo-inflammatory response. Yet nothing is known about the magnitude of thrombo-inflammation in DM compared with smokers. Higher inflammatory and pro-coagulant activity may explain the accelerated disease course of PAD in DM. Current guidelines recommend the same standard of care treatment in practice, and do not yet support more intensive treatment in diabetes-related PAD, which may require a combination of anti-thrombotic and anti-inflammatory drugs. Timing, optimal combination therapy, dosing and patient selection are some of the items that need to be clarified in future studies to ensure safe and successful treatment of PAD in DM.

## Data Availability

not applicable.

## List of abbreviations

α2AP: α2-antiplasmin
AGEs: advanced glycation end products
CCL2: CC-chemokine ligand 2
CLTI: critical limb threatening ischemia
CRP: C-reactive protein
DAPT: dual antiplatelet therapy
DM: diabetes mellitus
DNase: deoxyribonuclease
DOAC: direct-acting oral anticoagulants
FXIII: factor XIII
IKKβ: IĶB kinaseβ
IL-6: interleukin-6
JNK: JUN N-terminal kinase
NETs: neutrophil extracellular traps
NNT: number needed to treat
NO: nitric oxide
PAD: peripheral artery disease
PAI: plasminogen activator inhibitor
PKC: protein kinase C
ROS: reactive oxygen species
TF: tissue factor
TFPI: tissue factor pathway inhibitor
TNF-α: tumor necrosis factor-α
tPA: tissue plasminogen activator
uPA: urokinase-type plasminogen activator
vWF: von Willebrand Factor
